# The effects of operative stress in abdominal surgery on portal and arterial visceral circulation

**DOI:** 10.3389/fphys.2026.1804292

**Published:** 2026-04-27

**Authors:** Vid Pivec, Andrej Bergauer, Karin Schmid-Zalaudek, Nandu Goswami

**Affiliations:** 1 Clinical Department for Abdominal and General Surgery, University Clinical Centre Maribor, Maribor, Slovenia; 2 Research Unit of Gravitational Physiology and Medicine, Division of Physiology and Pathophysiology, Otto Loewi Research Center for Vascular Biology, Immunology and Inflammation, Medical University of Graz, Graz, Austria; 3 Department for General and Visceral Surgery, LKH Südweststeiermark, Standort Wagna, Wagna, Austria; 4 Center for Space and Aviation Health, College of Medicine, Mohammed Bin Rashid University of Medicine and Health Sciences, Dubai Health, Dubai, United Arab Emirates

**Keywords:** reactive hyperemia, postoperative/post injury splanchnic hyperemia, reactive-like hyperemia, isolated abdominal injury, portal vein, doppler ultrasound, splanchnic circulation, network physiology

## Abstract

Reactive hyperemia of the splanchnic circulation after isolated intra-abdominal organ injury has not been demonstrated in humans. Characterizing this response is essential for understanding postoperative hemodynamics and for validating non-invasive monitoring approaches. The aim of this study was to characterize the splanchnic hemodynamic response to isolated intra-abdominal organ injury and to assess the reliability of Doppler ultrasound for quantifying portal and visceral blood flow in this specific clinical setting. In this prospective observational study, 60 patients were enrolled into three groups: right hemicolectomy (n = 20), radical gastrectomy (n = 20), and ventral hernia repair without organ resection (n = 20). Elective surgery served as a standardized model of isolated intra-abdominal organ injury, with hernia repair as the control condition. Doppler ultrasound was performed preoperatively and daily for five postoperative days to assess flow and diameter of the portal vein (PV), superior mesenteric artery, celiac trunk, and abdominal aorta. Portal venous flow was indexed to body surface area and expressed as the portal flow index (VPI/BSA). Data were analyzed using repeated-measures ANOVA and McNemar’s tests. Portal venous flow was successfully obtained in all patients (age 67.4 ± 11.34 years; height 169.25 ± 7.69 cm; males n = 37, females n = 23). Arterial vessel visibility declined significantly over time (p < 0.01). A significant effect of time on ΔVPI/BSA was observed (F(5,53) = 15.99, p < 0.001, ηp² = 0.601), with a significant interaction between surgical group and time (F(10,108) = 5.04, p < 0.001). Right hemicolectomy and gastrectomy were associated with a transient increase in portal flow of up to 21% from baseline values, peaking on postoperative day 1, while minimal changes were observed in controls. PV diameter did not differ significantly between groups, although temporal patterns varied. Standardized isolated intra-abdominal organ injury therefore induces a reproducible, reactive hyperemia–like response in the portal circulation, and Doppler ultrasound represents a reliable, non-invasive modality for monitoring portal venous flow in this context. Arterial assessment is limited postoperatively. These findings provide physiological evidence of reactive splanchnic hyperemia in humans and support the clinical utility of portal flow monitoring after abdominal surgery.

## 1 Introduction

When tissue or organ damage occurs, inflammatory mediators and growth factors induce localized hyperemia. This transient rise in blood flow removes harmful substances, supports repair, and restores homeostasis. The hepatovisceral circulation, especially the portal system, may act as a temporary “blood donor” during major injuries, hypotension, or shock of various etiologies (Malbrain et al., 2004). Numerous studies have described reductions in portal blood flow under systemic stressors such as intensive exercise (Osada et al., 1999; Osada et al., 2009; Thijssen et al., 2009; van Wijck et al., 2012; Knight et al., 2017), sepsis (Malbrain et al., 2004; Takala, 1996; Schmidt and Stallmach, 2007), severe burns (Carter et al., 1988; Holm et al., 2006), heat stress (Crandall and Wilson, 2015), polytrauma (Wilmore et al., 1980; Gottlieb et al., 1984; Malbrain et al., 2004), hypovolemic shock (Krejci et al., 2000; Holm et al., 2006; Hinghofer-Szalkay et al., 2008), and thoracic epidural analgesia (Krejci et al., 2000; Gould et al., 2022; Hogan et al., 1993). These share a common mechanism: sympathetic activation with mesenteric vasoconstriction, which redistributes visceral blood into the systemic circulation. While this systemic response is well established, the phenomenon of reactive hyperemia—well known in other tissues after transient ischemia or injury—has not yet been demonstrated in visceral organs or visceral circulation following isolated abdominal organ injury. This study addresses that gap.

This study is based on preliminary data from two pilot studies that were performed by our group. The first, presented at the 2019 Quadrilateral Physiology Symposium in Graz (Pivec and Bergauer), showed that Doppler ultrasound could reliably quantify changes in visceral and portal flows. In eight healthy men (Mean age 37.4 years, height 180 cm and 82.1 kg) strenuous exercise caused a marked reduction in portal vein diameter and flow. In the second pilot study, involving five patients (Mean age 65,3 years +/-7.4 SD, height 172.4cm +/- 10.5 SD, males N = 3, females N = 2) undergoing colon resection, portal and mesenteric flows increased by ~30% at 48–72 hours postoperatively, returning toward baseline in the following days. The data from these studies suggested a hyperemic response and this led us to investigate the visceral circulatory response to isolated abdominal organ injury and test whether reactive hyperemia occurs in this setting. The hypothesis that was tested is that:

Isolated abdominal organ trauma induces postinjury (reactive – like) hyperemia in the visceral circulation, characterized by a transient rise in blood flow that peaks within 48 hours and normalizes in the following days. In addition, investigated was also the reliability of Doppler ultrasound in estimating changes in blood flow through the portal vein (PV), superior mesenteric artery (SMA), celiac trunk (TC), and abdominal aorta (AA) after isolated abdominal organ injury.

## 2 Methods

This study was a collaboration between the Medical University of Graz, Austria, and University Medical Centre Maribor, Slovenia. The project was approved by the DocSchool Translational Molecular and Cellular Biosciences, MedUni Graz on September 11, 2019 (ID: mugthesis: 7289) and received ethical approval from the Slovenian Central Medical Ethics Committee on December 17, 2021 (Ref: 0120-504/2021/3).

### 2.1 Study design

This was a prospective observational study. Isolated abdominal organ injury was modeled by observing patients scheduled for elective right hemicolectomy and radical gastrectomy. Patients undergoing ventral hernia repair served as controls. In this group, no intra-abdominal organ resection was performed; however, all groups were exposed to general anesthesia and median laparotomy. Differences between the resection groups and controls were therefore assumed to reflect the effect of intra-abdominal organ injury.

### 2.2 Participants

Sixty patients were enrolled into three groups (N = 20 per group). Patients who had right hemicolectomy for carcinoma of the right colon or cecum, radical gastrectomy for gastric carcinoma (subtotal and partial procedures excluded) and ventral hernia repair without intra-abdominal resection.

The following inclusion criteria were used. Adults in the above categories, ASA class II–III, who provided informed written and signed consent. The exclusion criteria included: ASA > III, liver cirrhosis, inflammatory bowel disease, chronic kidney disease (stage ≥ 3), end-stage renal failure, heart failure (NYHA ≥ II), or neurological/psychiatric disorders impairing consent.

Only patients with an uneventful postoperative course or minor complications (Clavien–Dindo (Dindo et al., 2004) grade I) were included in the final analysis. Patients requiring blood transfusion, significant fluid resuscitation, vasopressor support, prolonged antibiotic therapy or reoperation were excluded, in order to minimize major systemic confounders.

### 2.3 Study protocol and procedures

Daily monitoring included vital signs (CVP three times daily, arterial pressure and heart rate hourly, urine output, and temperature) and routine laboratory tests (CBC, CRP, electrolytes, liver and kidney function, total protein). In addition Doppler ultrasound (Esaote MyLab 70™) was used to measure diameter and flow in the portal vein (PV), superior mesenteric artery (SMA), celiac trunk (TC), and suprarenal abdominal aorta (AA). Examinations were standardized (supine position, 30-min rest, ≥4 h postprandial). Each vessel was measured three times; mean values were analyzed. Flow volume was calculated as vessel cross-sectional area × time-averaged velocity using the system software.

Each vessel was measured three times, and mean values were used for analysis. All ultrasound measurements were performed in accordance with standard clinical Doppler ultrasound practice. The abdominal ultrasound probe was positioned in the right upper quadrant, with the portal vein visualized in-plane.. Only insonation angles ≤60° were accepted to ensure accurate velocity estimation. The pulsed-wave Doppler sample volume was positioned centrally within the vessel lumen, avoiding wall proximity, to capture a representative laminar flow profile. For portal vein (PV) flow assessment, a 10–15-second pulsed-wave (PW) Doppler recording representative of stable flow at the hepatic hilum (PV confluence) was manually selected and analyzed. At the same anatomical location, vessel diameter was measured, assuming a circular cross-section, to calculate vessel area. Based on these measurements, the ultrasound system’s integrated software calculated the time-averaged mean velocity (TAMV) and cross-sectional area, and derived portal venous flow volume (mL/min) as their product.

Portal venous flow was normalized to body size and expressed as the portal flow index (VPI/BSA), calculated as PV flow divided by body surface area (BSA). Body surface area was calculated using the DuBois and DuBois anthropometric formula (0.007184 × height 0.725 × weight0.425) (Du Bois and Du Bois, 1916). Temporal changes were expressed as ΔVPI/BSA, defined as the difference between postoperative and baseline (preoperative) values.

All measurements were performed by the principal investigator (consultant HPB surgeon) supervised by the second author (consultant vascular surgeon), both with advanced ultrasound expertise. Data were recorded electronically and in duplicate on paper.

### 2.4 Statistical analysis

To assess the usefulness of US in measuring postoperative visceral arterial flow McNemar’s chi-square tests were performed. To analyze the effect of time and surgery type on portal vein flow and diameter, a repeated measures ANOVA was conducted using the surgery group as the between and the day of measurement as the repeated measures factor. All data were analyzed by IBM SPSS (Statistics for Windows, Version 30.0. Armonk, NY: IBM Corp). Mauchly’s test of sphericity and Levene’s test homogeneity were performed to assess the assumptions of the analysis.

### 2.5 Normality testing

The Shapiro-Wilk test showed that most variables followed a normal distribution. However, age was negatively skewed in both the Right Hemicolectomy and Radical Gastrectomy groups (p < 0.05), meaning most patients were older with a few younger outliers. For other variables (Weight, Height, BSA, and BMI), parametric tests were appropriate as normality held.

## 3 Results

Data were collected from 60 patients across three surgical groups: Right Hemicolectomy (n = 20, 11 females, 9 males), Radical Gastrectomy (n = 20, 5 females, 15 males), and Ventral Incisional Hernia (n = 20, 7 females, 13 males). The analyzed anthropometric variables were weight (kg), height (cm), age (years), body surface area (BSA, m²), and body mass index (BMI, kg/m²). (**Table 1**).

**Table 1:** Anthropometric data and the associated descriptive Statistics.

Variables	Right hemicolectomy (Mean ± SD)	Radical gastrectomy (Mean ± SD)	Ventral incisional hernia (Mean ± SD)
Weight (kg)	74.14 ± 9.88	79.43 ± 17.53	90.45 ± 13.72
Height (cm)	164.9 ± 8.27	172.2 ± 9.64	170.65 ± 5.15
Age (years)	71.00 ± 12.77	62.85 ± 12.89	68.35 ± 8.63
BSA (m²)	1.83 ± 0.13	1.96 ± 0.24	2.06 ± 0.17
BMI (kg/m²)	27.46 ± 4.85	26.57 ± 4.64	31.07 ± 4.57
N	20	20	20

A one-way ANOVA was performed to compare the effect of surgical group (Right Hemicolectomy, Radical Gastrectomy, Ventral Incisional Hernia) on weight, height, BSA, and BMI. Significant differences between the gropus were found for all variables: weight (F(2,57)=7.00,p=0.02), height (F(2,57)=4.72,p=0.013) BSA (F(2,57)=7.79,p=0.001) and BMI (F(2,57)=5.16,p=0.009).

**Figure 1:**
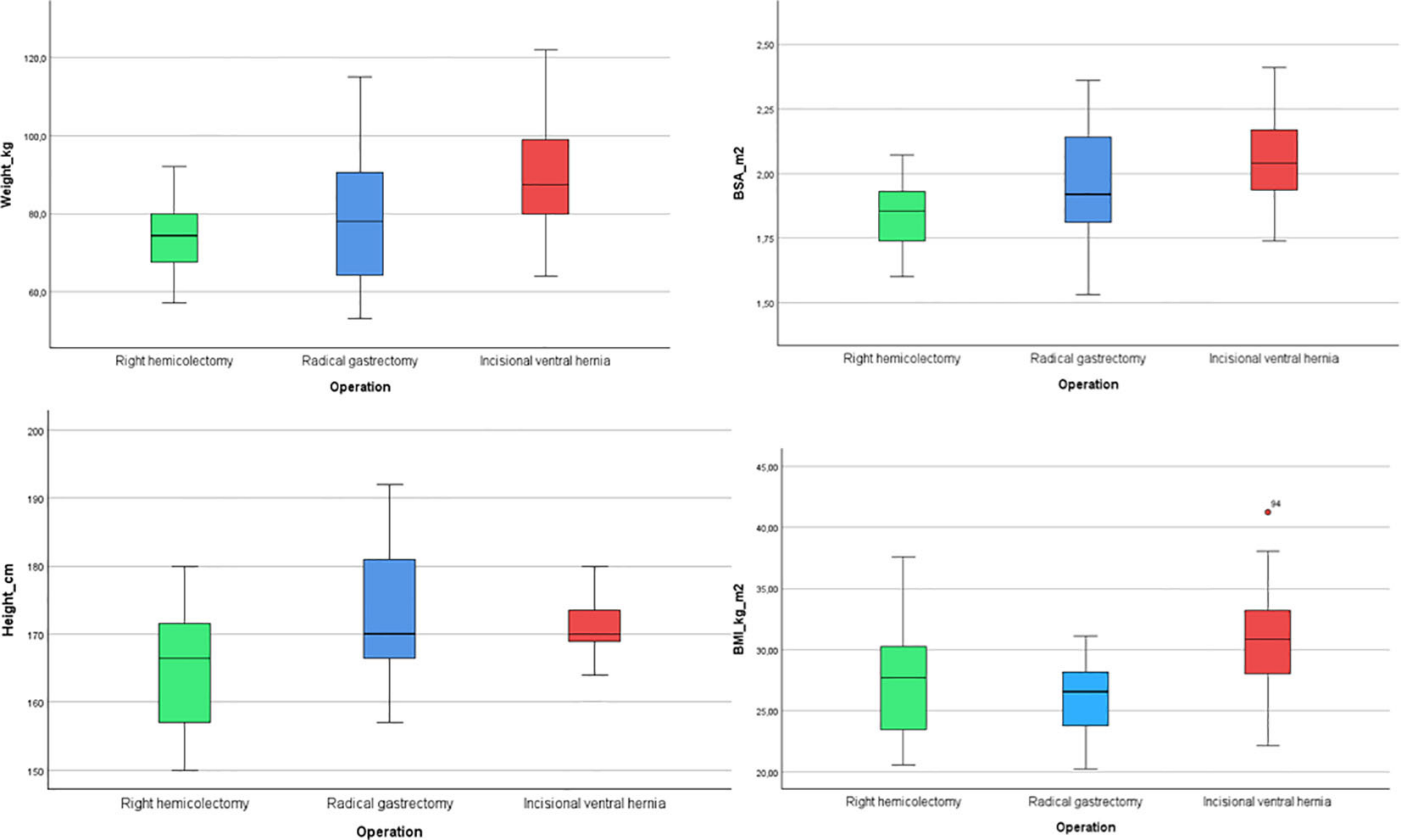
Comparison of anthropometric values across groups.

Weight and BSA: were significantly higher in the Ventral Incisional Hernia group compared to the other two groups (p <.05). (**Figure 1**). With respect to height: The Right Hemicolectomy group was an aware smaller than the Radical Gastrectomy group (p <.05). While with respect to the BMI: The Ventral Incisional Hernia group had a higher BMI than the Radical Gastrectomy group (p <.05). These findings suggest that patients in the Ventral Incisional Hernia group tend to have higher body weight and BMI.

To evaluate the usefulness of ultrasound (US) in the context of postoperative splanchnic vascular flow measurements, a McNemar test was conducted to assess whether there were statistically significant changes in the measurability of the abdominal aorta (AA), superior mesenteric artery (SMA), and truncus coeliacus (TC). The portal vein (PV) was visible in all analyzed patients and so its usefulness was not questioned in this analysis (**Table 2**).

**Table 2:** Preoperative PV flow index (absolute values).

Right hemicolectomy	Radical gastrectomy	Ventral hernia
490.29 ± 128.52 ml/min/m²	480.16 ± 141.15 ml/min/m²	459.68 ± 114.91 ml/min/m²

### 3.1 Results of the McNemar tests were as follows

AA: There was a statistically significant difference in measurability between Day 0 and Day 5, p<.001, indicating a significant change over time.
TC: There was a statistically significant difference in measurability between Day 0 and Day 5, p=.002.
SMA: There was a statistically significant difference in measurability between Day 0 and Day 5, p<.001.

**Figure 2:**
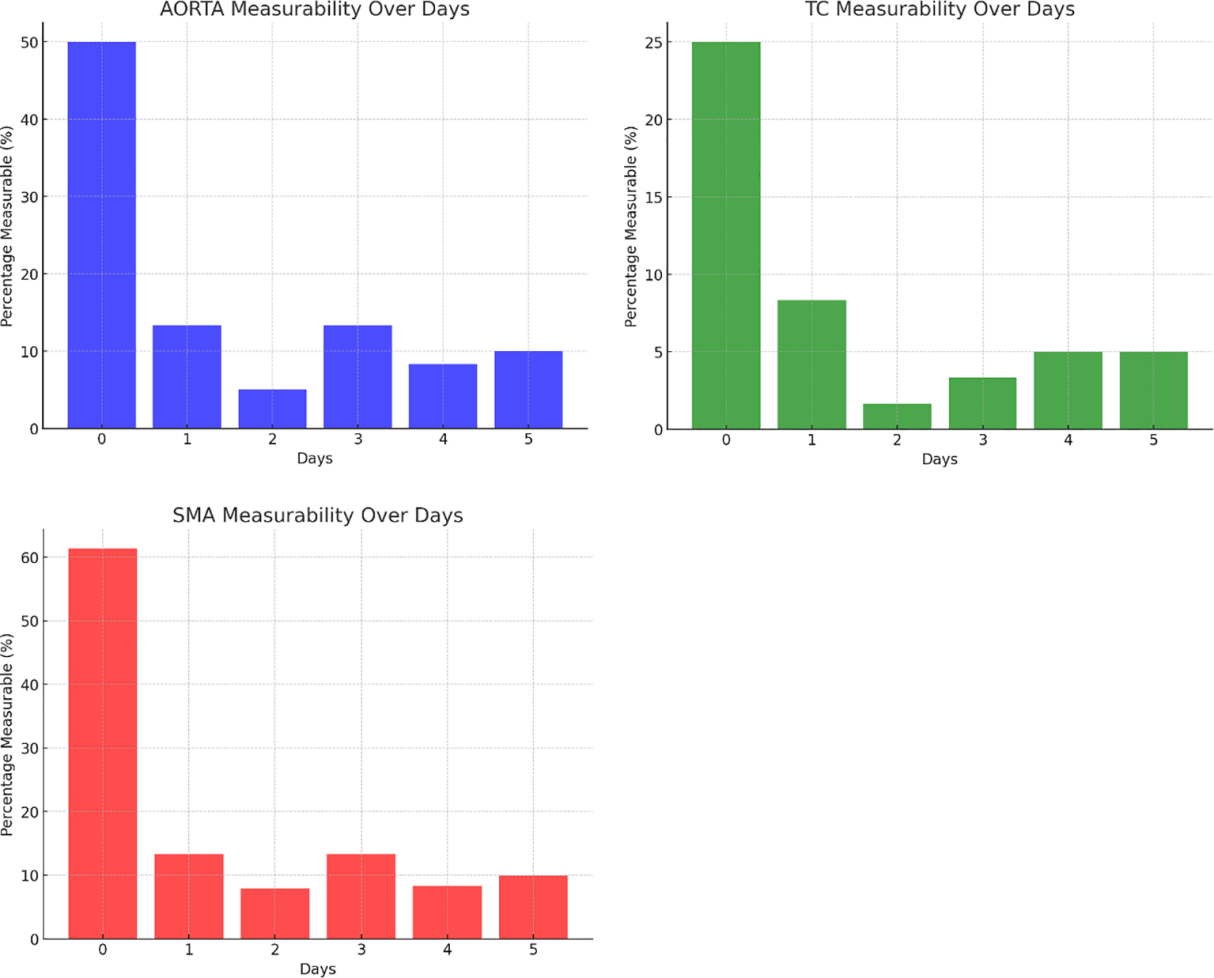
Percentage of vessel visibility over time.

These results suggest that the measurability of these vessels significantly decreased from Day 0 to Day 5 across all three groups (AA, TC, and SMA, **Figure 2**).

### 3.2 Portal venous flow

To evaluate the effects of time and surgery on changes in portal vein (PV) diameter and flow, a repeated measures ANOVA for selected parameters was performed: portal vein diameter (VP2r) and the daily differences of the portal flow index (ΔVPI/BSA). ΔVPI/BSA was chosen over direct flow measurements to adjust for body size differences between participants. Prior to the analyses, normal distribution was checked using Shapiro-Wilk test, while homogeneity of variances was tested by Levene’s test. *Post-hoc* analyses were performed for pairwise comparisons.

A two-way repeated measures ANOVA revealed a significant main effect of time on ΔVPI/BSA, (F(5, 53) = 15.99, p <.001, ηp² = .601), indicating substantial changes in portal venous flow across postoperative days. The interaction between time and surgery type was also significant, (F(10, 108) = 5.04, p <.001, ηp² = .318), suggesting the pattern of change differed by surgery type.

Time accounted for 60.1% of the variance in ΔVPI/BSA (Pillai’s Trace = .601, F(5, 53) = 15.99, p <.001, ηp² = .601) (**Table 3**). There was also a significant interaction between time and surgery type (Pillai’s Trace = .637, F(10, 108) = 5.04, p <.001, ηp² = .318), indicating that the change in ΔVPI/BSA over time varied by surgery type. *Post-hoc* tests showed these differences were most pronounced on the 1st and 2nd postoperative days, in patients who underwent radical gastrectomy and right hemicolectomy.

**Table 3:** PV flow index over time (relative values).

ΔVPI/BSA through time ml/minm^2^				
Measurement number	ΔVPI/BSA through time	Radical gastrectomy	Right hemicolectomy	Ventral incision hernia
1	Preoperative ΔVPI/BSA Index	-0.0 ± 0.0	-0.0 ± 0.0	-0.0 ± 0.0
2	Day 1 ΔVPI/BSA Index	79.45 ± 72.11	100.55 ± 75.21	-9.8 ± 27.74
3	Day 2 ΔVPI/BSA Index	71.98 ± 48.82	55.68 ± 83.97	-17.25± 34.34
4	Day 3 ΔVPI/BSA Index	9.08 ± 66.84	30.04 ± 60.99	-27.38 ± 39.0
5	Day 4 ΔVPI/BSA Index	-7.16 ± 45.79	15.96 ± 46.72	-12.48 ± 36.22
6	Day 5 ΔVPI/BSA Index	-0.89 ± 40.3	-5.69 ± 36.39	-8.91 ± 26.29

**Figure 3:**
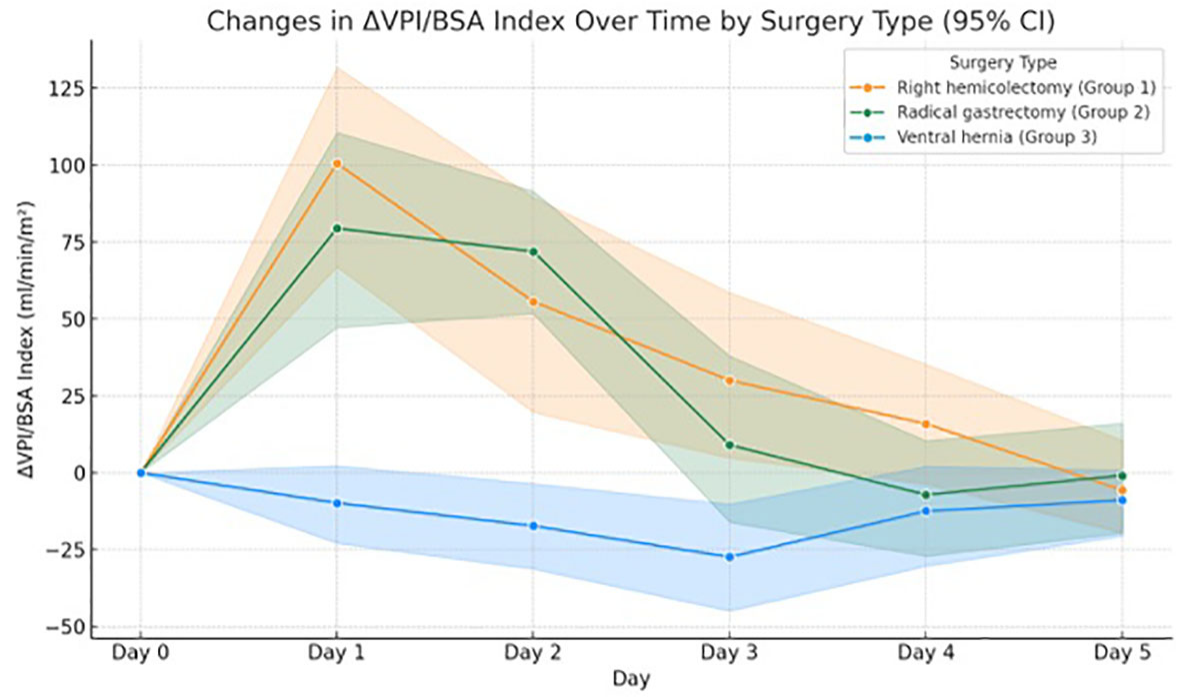
Changes of relative PV flow index over time.

Tukey’s HSD test revealed significant differences in ΔVPI/BSA on postoperative Day 1 between the right hemicolectomy and ventral hernia groups (mean difference = 45.39, p <.001) and between the radical gastrectomy and ventral hernia groups (mean difference = 38.05, p = .001). These differences persisted on Day 2 for the same groups. No significant difference was found between the right hemicolectomy and radical gastrectomy groups (mean difference = 7.35, p = .752) (**Figure 3**).

### 3.3 Results for vessel diameter

**Figure 4:**
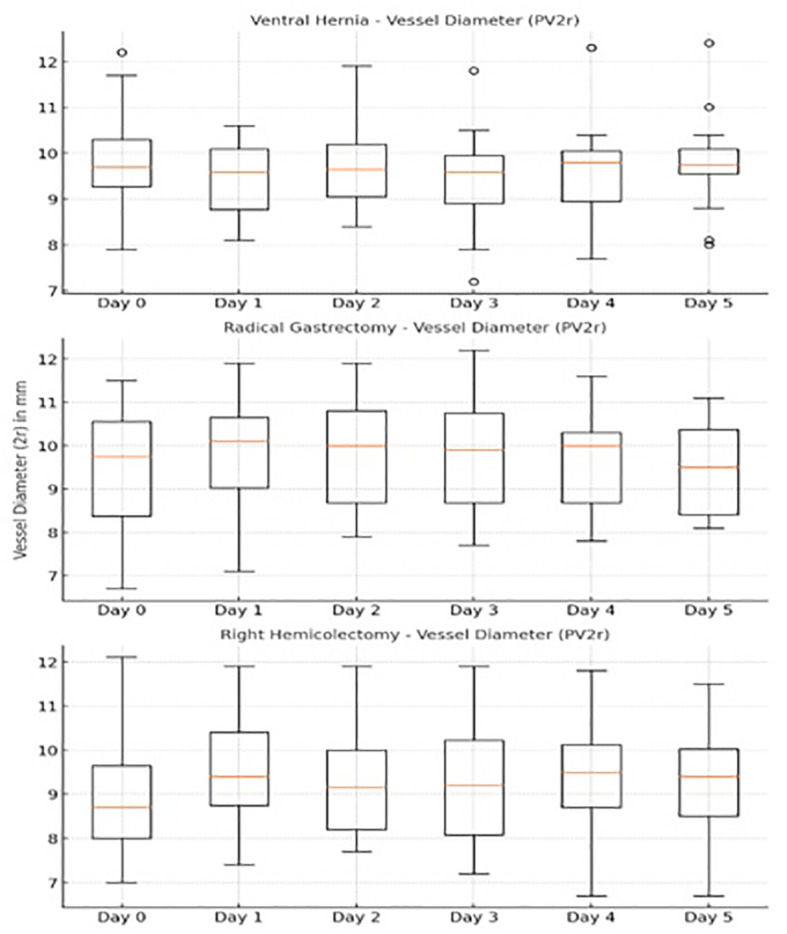
Changes of PV vessel diameter over time.

Also a repeated-measures ANOVA was conducted to examine the effect of time (Day 0 to Day 5) and surgery type (Ventral Hernia, Right Hemicolectomy, and Radical Gastrectomy) on vessel diameter (PV2r). (**Figure 4**). Mauchly’s test indicated that the assumption of sphericity was violated for the main effect of time, therfore the Greenhouse--Geisser correction was applied to the degrees of freedom for the repeated-measures ANOVA.

No significant main effect of time on vessel diameter, F(4.05,230.87)=1.30, p=.271 was found. However there was a significant interaction between time and surgery type, (F(8.10,230.87)=2.85,p=.005) indicating that changes in vessel diameter over time differed by surgery type. Pairwise comparisons with Bonferroni correction revealed no statistically significant differences in overall mean vessel diameters between surgery types, (p>.05) An interaction plot showed that while the overall mean vessel diameters did not differ significantly between surgery types, the patterns of change over time were distinct. Surgery type 3 (Ventral Hernia) showed more stability, while surgery type 1 (Right Hemicolectomy) exhibited greater variability.

## 4 Discussion

In this prospective observational study of elective abdominal surgery, we demonstrate that isolated intra-abdominal organ injury induces a reproducible, time-dependent increase in portal venous blood flow, consistent with a reactive hyperemia like response of the splanchnic circulation. Doppler ultrasound enabled reliable longitudinal assessment of portal venous flow across the postoperative period, whereas serial evaluation of visceral arterial vessels proved increasingly limited. After adjustment for intergroup differences in body size using an indexed portal flow parameter (VPI/BSA), a significant main effect of time and a strong interaction between time and surgery type were observed. Right hemicolectomy and radical gastrectomy were associated with a transient increase in portal venous flow of up to approximately 20% above baseline, peaking on postoperative days 1, while only minimal changes were observed in the ventral hernia control group. Portal vein diameter did not change uniformly over time, indicating that the observed hyperemia was primarily flow-driven rather than attributable to consistent venous dilatation. In contrast, postoperative measurability of the abdominal aorta, celiac trunk, and superior mesenteric artery declined significantly, underscoring technical limitations of arterial Doppler assessment in the early postoperative setting.

To the best of our knowledge, this is the first study to specifically characterize the visceral vascular response to isolated abdominal organ injury in humans. Previous investigations have predominantly focused on visceral hemodynamic responses to systemic stressors such as sepsis, exercise, burns, polytrauma, or shock, leaving the localized splanchnic response to abdominal trauma largely unexplored. By using elective surgery as a controlled and standardized model of isolated abdominal injury, the present study addresses this important gap and introduces a novel methodological approach to studying human visceral vascular physiology.

With respect to the first primary endpoint, our findings clearly demonstrate that Doppler ultrasound is a reliable and practical tool for measuring portal venous flow and diameter in the perioperative and postoperative setting. The absolute preoperative portal venous flow values and portal vein diameters observed in our cohort were consistent with established physiological ranges reported in the literature (Huang et al., 2023). In line with this, Wilmor DW et al. (Wilmore et al., 1980) reported that normal resting total hepatic blood flow (THBF) ranges between 630 and 850 mL/min/m². Assuming that approximately 80% of THBF is attributable to portal venous inflow, this corresponds to an estimated resting portal flow index (VPI) of approximately 500–680 mL/min/m². These literature-derived values are in good agreement with our measurements, further supporting their validity. Furthermore, the ability to detect significant time-dependent changes in portal flow confirms the sensitivity of Doppler ultrasound for longitudinal assessment, provided the examination is performed by operators with adequate ultrasound expertise (Jäger et al., 1986). In contrast, arterial measurements were substantially limited by declining postoperative visibility, with statistically significant reductions in measurability for the abdominal aorta, celiac trunk, and superior mesenteric artery. Due to the progressive decline in postoperative vessel measurability, our data do not allow robust conclusions regarding arterial flow dynamics. The observed reduction in arterial visibility should therefore be interpreted primarily as a feasibility limitation rather than a definitive assessment of Doppler ultrasound performance in this setting.

The second primary endpoint—the demonstration of a transient postoperative increase in portal venous flow following visceral organ resection—represents a novel physiological observation. The significant interaction between time and surgery type indicates that the pattern of portal flow recovery depends on the nature of the surgical intervention. Both right hemicolectomy and radical gastrectomy showed a pronounced hyperemic response within the first 48 postoperative hours, whereas the control group undergoing ventral hernia repair exhibited minimal change. This temporal and group-specific pattern strongly supports the hypothesis that isolated abdominal organ injury triggers a localized reactive hyperemia within the splanchnic circulation, rather than reflecting nonspecific effects of anesthesia, laparotomy, or perioperative stress alone. An important consideration is that the observed increase in portal venous flow may not exclusively reflect a localized hyperemic response but could also be influenced by systemic hemodynamic changes, including variations in cardiac output, perioperative fluid administration, and inflammatory responses.

However, the absence of a comparable increase in the control group, which was exposed to similar anesthetic and surgical conditions without organ resection, suggests that local factors related to organ injury may play an important role. Therefore, the observed portal flow changes likely reflect a combination of local and systemic mechanisms, with local factors related to organ injury potentially playing a predominant role.

### 4.1 Possible mechanisms of postoperative splanchnic hyperemia

The splanchnic circulation is a highly dynamic vascular bed that plays a central role in systemic hemodynamic regulation, acting as a major blood reservoir and contributing substantially to total vascular capacitance (Brooksby and Donald, 1971). Its perfusion is governed by an integrated hierarchy of neural, humoral, endothelial, and local metabolic mechanisms that continuously adapt regional blood flow to both systemic demands and local tissue requirements.

At the central and neural level, the splanchnic vascular bed is under strong sympathetic control, with norepinephrine-mediated activation of α-adrenergic receptors leading to vasoconstriction and redistribution of blood flow away from the splanchnic region during stress conditions such as hemorrhage or surgery (Takala, 1996; Brooksby and Donald, 1971). Despite this, a phenomenon of “autoregulatory escape” allows partial restoration of flow at the microcirculatory level, thereby protecting intestinal perfusion from sustained ischemia (Krejci et al., 2000).

At the humoral level, circulating vasoactive hormones such as angiotensin II and vasopressin further increase mesenteric vascular resistance during hypovolemia and systemic stress, whereas postprandial and metabolic signals can promote vasodilatation.

Endothelial and paracrine mechanisms provide an additional level of fine regulation. Nitric oxide (NO), prostacyclin (PGI₂), and carbon monoxide (CO) act as key vasodilators through smooth muscle relaxation, whereas endothelin-1 (ET-1) exerts potent vasoconstrictive effects, particularly in the context of tissue injury and inflammation (Koeppen, 2023a; Koeppen, 2023b).

Finally, local metabolic regulation plays a crucial role in matching perfusion to tissue demand. Accumulation of metabolites such as adenosine, hydrogen ions (pH changes), and carbon dioxide (CO₂) during hypoxia or reduced perfusion induces vasodilatation of resistance vessels, facilitating restoration of oxygen delivery. These mechanisms are central to the development of reactive hyperemia, defined as the transient increase in blood flow following a period of reduced perfusion (Koeppen, 2023a; Koeppen, 2023b).

Within this physiological framework, the postoperative increase in portal venous flow observed in the present study can be interpreted as a multifactorial response involving both local and systemic mechanisms. One important contributor is likely reactive hyperemia following transient intraoperative hypoperfusion (Gould et al., 2002). Experimental human data have demonstrated that reduction in splanchnic perfusion (Rosenberry and Nelson, 2020), such as during central hypovolemia, is followed by a significant rebound increase in hepatic blood flow upon restoration of normal conditions, reflecting autoregulatory vasodilatation (Hinghofer-Szalkayet al., 2008).

In addition, surgical trauma and tissue manipulation induce a complex inflammatory response associated with increased metabolic activity and release of vasoactive mediators, which may further promote regional vasodilatation and increased splanchnic blood flow . These mechanisms are likely amplified by local endothelial activation and increased production of vasodilatory substances such as nitric oxide.

Furthermore, the splanchnic circulation is highly sensitive to systemic stress and redistribution phenomena. A reduction in sympathetic tone following intraoperative stress may contribute to a transient overshoot in perfusion (“post-stimulation hyperemia”), while the dissociation between systemic and regional perfusion has been well documented, indicating that changes in splanchnic blood flow may not directly parallel global hemodynamic parameters15.

Taken together, these findings suggest that the observed postoperative increase in portal flow likely reflects a combination of reactive hyperemia, inflammation-driven vasodilatation, and systemic hemodynamic adjustments, rather than a single isolated mechanism.

### 4.2 Clinical implications

These findings have several clinically relevant implications. At present, there are no inexpensive, repeatable bedside methods for obtaining objective, quantitative information about intra-abdominal organ perfusion, apart from intra-abdominal pressure (IAP) measurements. While clinicians routinely monitor cardiovascular and pulmonary status using parameters such as central venous pressure, electrocardiography, heart rate, respiratory rate, and oxygen saturation, comparable objective markers for abdominal organ perfusion are lacking. Most commonly used abdominal assessments—such as tenderness, bowel sounds, or pain perception—remain subjective.

Introducing a measurable parameter such as portal venous flow, which can be readily taught to medical students and junior doctors, and performing repeated measurements throughout the day could therefore open new avenues for structured data collection. This approach would allow correlation of portal flow dynamics with other physiological processes and disease states. In the present study, the observed transient reactive - like hyperemia appears to represent a physiological marker of uncomplicated postoperative recovery. Deviations from this expected temporal pattern—either attenuation, delay, or exaggeration of the hyperemic response—may potentially signal evolving complications. A clearer understanding of the timing and magnitude of postoperative splanchnic hyperemia could thus contribute to more individualized postoperative management, particularly with respect to fluid therapy, hemodynamic support, and early detection of adverse recovery trajectories.

### 4.3 Limitations

Several limitations of this study should be acknowledged. First, the relatively small sample size and the exclusion of patients with severe comorbidities limit the generalizability of the findings to broader surgical populations. Second, the study groups were not fully homogeneous with respect to anthropometric characteristics; in particular, patients undergoing ventral hernia repair had a higher body mass index, whereas patients in the right hemicolectomy and radical gastrectomy groups were older. Although portal venous flow was indexed to body surface area to mitigate these differences, residual confounding cannot be fully excluded.

Third, systemic hemodynamic parameters such as cardiac output, detailed perioperative fluid balance, and stratification of patients according to the use of epidural analgesia were not systematically recorded. Although major systemic confounders were partially mitigated by excluding patients with postoperative complications above Clavien–Dindo grade I, this remains an important limitation that restricts the ability to fully distinguish between local and systemic contributors to the observed changes in portal venous flow.

Another limitation is the relatively short postoperative follow-up period, which precludes conclusions regarding the long-term physiological or clinical significance of the observed hyperemic response. The durability of these hemodynamic changes and their relationship to longer-term outcomes, such as delayed complications or recovery trajectories, remain unknown. Future studies should therefore aim to include larger and more heterogeneous patient cohorts, perform multiple daily flow measurements to better characterize short-term dynamics, and extend follow-up to evaluate the long-term implications of postoperative splanchnic hyperemia on recovery and complication rates.

## 5 Conclusions and future directions

In conclusion, this study suggests that localized abdominal trauma, such as that caused by surgery, induces a postoperative hyperemia in the splanchnic circulation. Additionally, we demonstrate that ultrasound is an effective tool for monitoring changes in portal venous blood flow, offering a reliable, non-invasive method for assessing visceral hemodynamics in the postoperative period. These findings could lead to improvements in postoperative monitoring and management of patients undergoing abdominal surgery. Future research should expand upon these results by investigating the long-term clinical outcomes associated with these hemodynamic changes.

## Data Availability

The datasets generated and analyzed during this study are not publicly available due to the presence of sensitive clinical data. Anonymized datasets can be made available upon reasonable request to the corresponding authors, in accordance with the EU General Data Protection Regulation and applicable institutional and ethical regulations.
